# TAL effectors: tools for DNA Targeting

**DOI:** 10.1093/bfgp/elu013

**Published:** 2014-06-06

**Authors:** Radek Jankele, Petr Svoboda

**Keywords:** TALE, TALEN, ZFN, FokI, DNA editing

## Abstract

*Xanthomonas* phytopathogenic bacteria produce unique transcription activator-like effector (TALE) proteins that recognize and activate specific plant promoters through a set of tandem repeats. A unique TALE-DNA-binding code uses two polymorphic amino acids in each repeat to mediate recognition of specific nucleotides. The order of repeats determines effector’s specificity toward the cognate nucleotide sequence of the sense DNA strand. Artificially designed TALE-DNA-binding domains fused to nuclease or activation and repressor domains provide an outstanding toolbox for targeted gene editing and gene regulation in research, biotechnology and gene therapy. Gene editing with custom-designed TALE nucleases (TALENs) extends the repertoire of targeted genome modifications across a broad spectrum of organisms ranging from plants and insect to mammals.

## INTRODUCTION

Engineered DNA-binding domains (DBDs) fused with different catalytic or effector domains allow researchers to edit DNA sequences or regulate gene expression at specific DNA loci within complex eukaryotic genomes. There are two main classes of engineered site-specific DBDs: zinc finger-based DBDs and transcription activator-like effector (TALE)-based DBDs. Site-specific zinc finger nucleases (ZFN) for genome editing (reviewed in [1]) pawed the road for the TALE Nuclease (TALEN) technology, which is based on a unique modular DBD of TALEs from plant-pathogenic bacterial genus *Xanthomonas.* A commonly used nuclease domain in ZNFs and TALENs is the dimerizing FokI endonuclease cleavage domain, which introduces a double-strand break (DSB) [2, 3]. DSBs at targeted loci rapidly increase local frequencies of homologous recombination. This enables the extension of genetic manipulations to virtually any model organisms and cell line.

In this review, we first recapitulate discovery of TALEs and deciphering of their binding code. Next, we describe the structure of TALE DBD and its implications for biotechnology. Finally, we discuss TALE-based nucleases and genome regulators as distinct categories of engineered site-specific proteins that share a common DBD but differ in their effector domains, hence in their mode of action.

## TALEs—VIRULENCE FACTORS OF *XANTHOMONAS*

Gram-negative γ-proteobacteria of the genus *Xanthomonas* are important plant pathogens affecting worldwide yields of crop plants such as wheat, rice, cassava or cotton. *Xanthomonas* enter host plants through surface wounds or natural openings and multiply inside plant tissues (reviewed in detail in [4]). To facilitate a productive bacterial infection in plants, *Xanthomonas* secrete a cocktail of effector proteins into host cells, including the TALE family proteins (originally denoted *AvrBs3*-family effectors) that function as eukaryotic-like transcription factors. TALEs are secreted directly into the plant cell cytoplasm [5] and transported into the nucleus via importin-α [6]. Recognition of specific promoters and subsequent interaction with the basal transcriptional machinery induce transcription of specific host plant genes.

**Figure 1:**
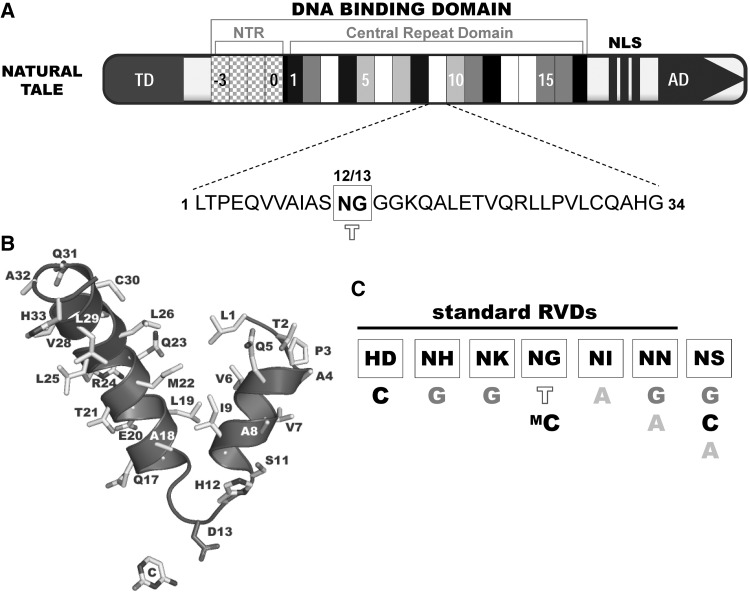
TALE domain composition and DNA-binding code. (**A**) TALEs contain nuclear localization signals (NLS) and an activation domain (AD) to function as transcriptional activators. A central tandem repeat domain confers specific DNA-binding and host specificity. Translocation signal (TD) and four cryptic repeats required for initiation of DNA binding and for the recognition of 5′-T^0^ are located at the N-terminus (chequered rectangles). Each 34 amino acid (aa) long repeat in the CRD binds to one nucleotide with specificity determined mainly by aa at position 13. One sample repeat is shown below the protein scheme. Numbers 12/13 refer to aa positions within the repeat. (**B**) Structure of an individual TALE repeat module. The repeat has 34 amino acids in length and takes a loop–helix secondary structure where two α-helices are linked by short ‘RVD loop’. The residue 13 is responsible for preferential binding of the repeat module to a single specific nucleotide in the major groove of target DNA sequence (C, in this case). (**C**) Repeat types have specificity for one or several nucleotides. Only bases of the DNA leading strand are shown. Adapted from [7, 9, 10, 11].

TALEs exhibit exceptional DNA-binding specificity stemming from a unique domain organization [7, 8] (Figure 1A). The common feature of natural TALEs is their DBD composed of 7–34 highly homologous direct repeats in the central part of the protein [9]. Typically, each repeat module (Figure 1B) has 34 amino acids (aa) in length; the last C-terminal truncated repeat, so-called half-repeat, consists of 20 aa. Two polymorphic aa residues at positions 12 and 13 form the repeat-variable diresidue (RVD), where the residue 13 is responsible for preferential binding of the repeat module to a single specific nucleotide in the major groove of target DNA sequence (summarized in [10]). The binding code was deciphered independently in 2009 by two groups who found a simple cipher, where common RVDs HD, NG/HG and NI recognize almost exclusively cytosine, thymine and adenine, respectively; whereas NN or NS has more degenerated specificity [7, 8] (Figure 1C). The order of repeat modules from N to C-terminus within TALE DBD then corresponds to the recognized DNA sequence in 5′ to 3′ direction such that each repeat contacts one specific DNA base pair via the RVD.

While the TALE central repeat domain (CRD) determines the specificity, the DBD is further extended ∼150 aa into the N-terminal region (NTR), immediately preceding the first canonical repeat [12–14]. This region is composed of four cryptic repeats and substantially contributes to the overall basic charge of TALE proteins [12, 15]. The NTR is necessary for binding of TALEs to DNA and mediates interaction with a conserved thymine at position 0 (discussed in more detail later). The N-terminus of natural TALE proteins also contains secretion and translocation signals required for delivery into host cells [16]. The C-terminal region carries conserved three monopartite nuclear localization signals and a conserved eukaryotic-like acidic transcriptional activation domain [6, 17–19]. Notably, TALE-like proteins were also identified in the plant pathogenic bacterium *Ralstonia solanacearum* [20, 21] offering additional options for engineering DBDs.

## SPECIFICITY OF DNA BINDING BY TALEs

**Figure 2:**
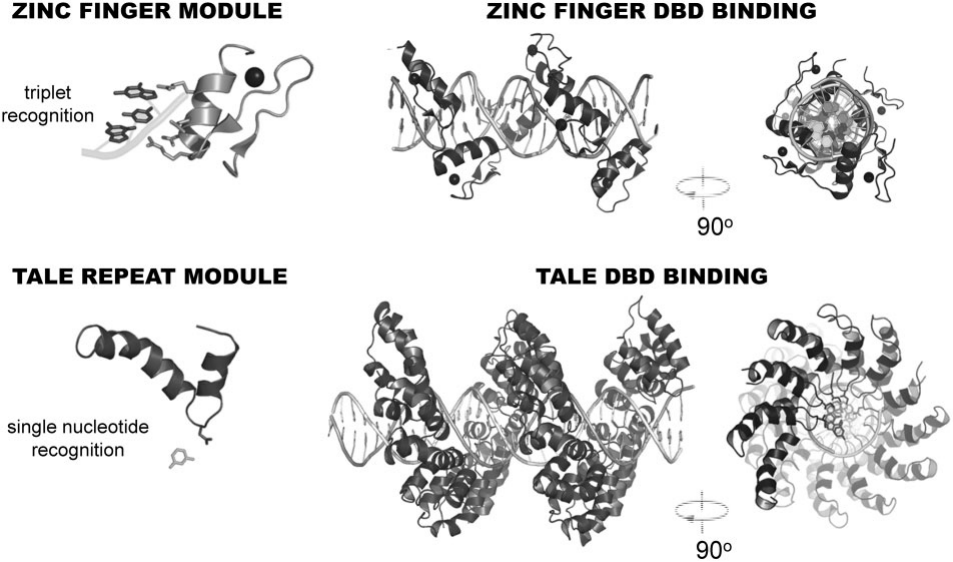
Comparison of zinc finger and TALE DNA-binding domains. A single zinc finger module recognizes three nucleotides of DNA while a TALE repeat module recognizes a single nucleotide of DNA. Next are shown front and lateral views of zinc finger and TALE DBDs. Shown is a six-finger zinc finger protein that consists of six tandem repeats of C2H2 zinc finger motifs, each consisting of approximately 30 amino acids and a TALE DBD consisting of 2 cryptic repeats and 22 canonical repeat modules. Structures were rendered using available structural information deposited in the Protein Data Bank [15, 22].

Crystallographic studies of TALEs bound to their target sequences unraveled that TALE DBD forms a right-handed superhelical assembly wrapped around B-form DNA duplex (Figure 2) and explained specific repeat-nucleotide interactions [15, 23, 24]. Individual TALE repeats have helix–loop–helix secondary structure where two α-helices are linked by short ‘RVD loop’ (Figure 1B). The first short α-helix spans residues 3–11 and the longer bended second α-helix spans residues 15–33. The RVD loop of each TALE repeat reaches into the major groove of the DNA duplex and contacts a single nucleotide in the sense strand with the residue at position 13 [15, 23]. Interestingly, the residue at position 12 (mainly histidine or asparagine) points away from the major groove and does not contribute to the specific base recognition but, rather stabilizes the position of the RVD loop [15, 23]. Within each repeat, lysine and glutamine residues at positions 16 and 17, respectively, contribute to non-specific interactions with negatively charged DNA backbone [15, 23]. The characteristic angle between inter-repeat helices distinguishes the TALE repeat domain from other known α-helical repeat domains [23].

### Recognition of nucleotides in the cognate sequence

Different types of interactions are responsible for recognition of different nucleotides. This is important for designing custom TALE domains. Direct H- bonds are involved in base selectivity for C, G, G/A and A/G/C mediated by RVDs HD, NH, NN and NS, respectively. Weaker van der Waals contacts are responsible for base selectivity of NI and NG for A and T, respectively [15,, 23 24]. Nucleotide-binding specificity is determined not only by possible contacts with nucleotides but also by steric exclusion of interactions with alternative nucleotides (reviewed in [25]). Notably, the use of HD and NG enables partial discrimination of targets with unmethylated or methylated cytosines with custom TALEs because NG can accommodate a methylated cytosine, whereas HD does not [15, 23]. In addition, a 33 aa long N* repeat (missing the residue at position 13) exhibits complete recognition promiscuity explained by absenting physical contact with nucleotides [15]. Therefore, N* also allows for accommodating methylated cytosines and for designing TALE domains with highly degenerated target specificity [26].

RVDs NI, HD, NH/NK and NG are highly specific, recognizing A, C, G and T nucleotides, respectively [7, 8]. NG and HD bind cognate bases with high, NH with ‘intermediate’ and NI and NK with weaker affinity [15, 27–29]. NN and NS have degenerated specificity; NN repeat selects both for G and A (with a preference for G) and binds them with high affinity [7, 27, 29–32]. NS can bind A, C and G; interaction with T is probably sterically excluded [7, 8, 15, 25]. Guanine is exclusively recognized by NK and NH [27, 29, 32, 33]. NH recognizes guanine with ‘intermediate’ affinity, whereas NK was classified as ‘weak’ and also performed poorly in reporter assays compared with both NN and NH [27, 29, 30, 32, 34, 35]. Thus, NH seems to be a good choice for G targeting, especially if flanked by a few strong RVDs (NG, NN and HD) [29, 32]. Repeats included in available TALE assembly kits (HD, NG, NI, NN, NH and NK) are further referred as standard RVDs, all other RVDs are referred as ‘non-standard’ (Figure 1C).

TALE-DNA-binding mechanism is apparently asymmetric across the protein–DNA interface [27]. NTR ensures 5′-T^0^ recognition and probably serves as a binding-anchor from which the protein wraps around a DNA helix and probes a nucleotides sequence [12]. Therefore, mutations at the 5′ end of a corresponding TALE target site impair activity more than mutations at the 3′ end [27, 31]. Furthermore, too many strong RVDs at the N-terminal part of CRD may pose a risk of multiple off-target effects. At the same time, weak RVDs at the C-terminal part of CRD may also impair TALE activity [27].

It seems that evolutionary optimal length of TALE arrays is between 17 and 20 RVDs, as most of natural TALEs fall within this range [9]. This possibly reflects a critical TALE size above which deformations in superhelical assembly could lead to registration errors. Thus, adding more repeats to an array may have no positive effect to overall binding affinity [9, 15, 27]. Moreover, a systematic study of TALEN specificity revealed that excess non-specific DNA-binding energy (which is increasing with an array length) results in tolerating more mismatches and, therefore, in greater off-target cleavage [31]. Accordingly, TALENs mutated at the C-terminal domain to reduce non-specific DNA-binding energy still retain high activity and exhibit improved specificity [31].

### The invariant 5′-thymine base

Interestingly, well-conserved thymine is present at the position 0 (T^0^) of most of natural TALE target sites [8] and is necessary for full target gene activation [7, 36] and activity of TALE fusion proteins [12, 37, 38]. Although structural data can explain the 5′-T^0^ preference [15], TALE fusion proteins functioning on 5′-T^0^-deficient target sites were also reported [27, 28, 39]. The significance of 5′-T^0^ differs for wild-type TALEs and artificial TALEs created with standard RVDs suggesting that the latter bind DNA with higher affinity and may not require the invariant 5’-T^0^ [27]. Recently, redesigned scaffolds allowing non-constrained target site selection were reported [38]. However, it is advisable to design artificial TALEs with 5’-T^0^, as this natural TALE’s feature does not seriously constrain target site selection in eukaryotic genomes.

## USE OF TALE DBD FOR GENE EDITING AND REGULATION

In their pioneering work, Boch *et al.* [7] demonstrated that artificial TALEs could be synthesized, hence allowing for exploitation of the TALE-binding code for targeting almost any DNA sequence with artificial TALE DBDs. Properties of the TALE DBD offer a great potential for research, biotechnology and gene therapy. Repeat modules can be arranged in a desired order to produce a DBD with high sequence specificity. Such a DBD can be combined with a catalytic or effector domain, e.g. a nuclease to obtain an exceptional tool for DNA editing [40]. High specificity, reliable activity and low cytotoxicity are desired features of an ideal customized nuclease.

TALE fusion proteins use the C-terminal region downstream of CRD as a linker between TALE DBD and the effector domain. The optimal length of the linker may vary for different effector domains, e.g. a short 17–65 aa linker is used for the dimerizing FokI nuclease domain [13, 28], whereas a longer linker (∼65 aa) was used for activation domains [14, 28]. This difference likely reflects different steric requirements of particular effector domains.

### Gene editing with TALE nucleases

Organisms repair DSBs through two major pathways: non-homologous end joining (NHEJ) and homologous recombination (HR). NHEJ is an error-prone process, which often leads to small insertions or deletions (indels) at the break site, and thus can cause a frameshift mutation in the coding sequence of targeted gene. HR is generally an error-free process, which can use a sister chromatid or exogenous homologous template to repair the damage. Traditional gene targeting relies on DSB-independent HR to replace (knock-in) or disrupt (knock-out) gene sequences in a pre-determined locus (reviewed in [41]). Low frequency of DSB-independent HR limits this approach to just a few model organisms (e.g. *Mus musculus,* or *Saccharomyces cerevisiae*) and cell types (e.g. embryonic stem cells). Even in suitable cells, the frequency of HR with the donor sequence is low (1/10^4^^–^^7^), requiring some selection system to identify cells where HR occurred. A remedy for this problem represents nuclease-induced DSBs, which stimulate HR [42, 43]. This nuclease-mediated approach is referred to as gene editing.

**Figure 3:**
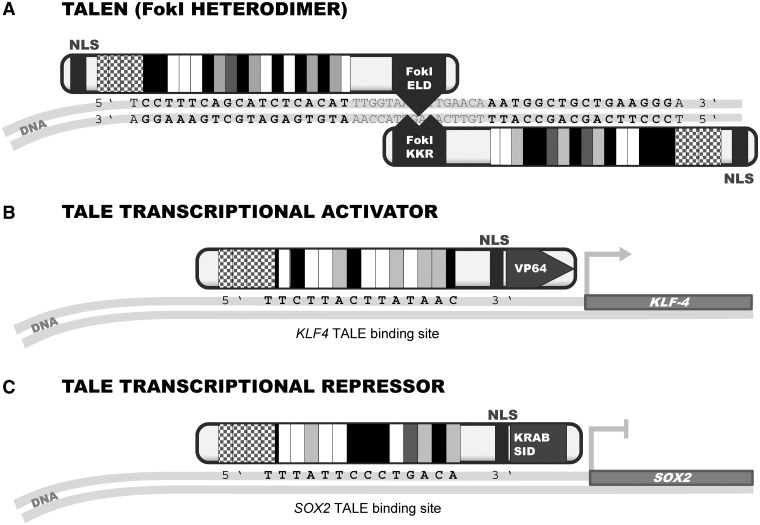
TALE-based gene editors and regulators. (**A**) A pair of TALENs with a heterodimerizing FokI domain [47]. (**B**) A TALE-based transcriptional activator [14]. (**C**) A TALE-based transcriptional repressor [32].

One of the first tools for gene editing was synthetic ZFN (Figure 2). A ZFN is created by linking the FokI nuclease domain [44] to a Cis_2_His_2_ zinc-finger array, which provides the sequence specificity [3]. The FokI nuclease domain functions as a dimer [2]; therefore, two zinc-finger arrays, each carrying a FokI monomer, are targeted to neighboring sites between which FokI dimerization occurs [1]. ZFN technology yielded substantial achievements in a variety of model organisms and cell types, which were previously inaccessible by the classical gene targeting methods. In contrast to traditional gene targeting, gene editing with custom nucleases yields high mutation frequencies; therefore, selectable markers are not necessary. Principles established during more than a decade of ZFNs research were subsequently adapted to TALENs once the TALE-DNA-binding code was deciphered. In TALENs, the FokI nuclease (or its heterodimeric variants [45, 46], Figure 3A) is recruited to two adjacent target sites separated with a short spacer (12–20 nt) (reviewed in [48]). In contrast to a zinc-finger DBD, where one finger predominantly recognizes a nucleotide triplet [49], each module of TALE DBD recognizes a single nucleotide within the target sequence (Figure 2). The initial TALEN fusions with the homodimeric FokI demonstrated successful TALEN-mediated alterations [28, 37, 40, 50].

**Figure 4:**
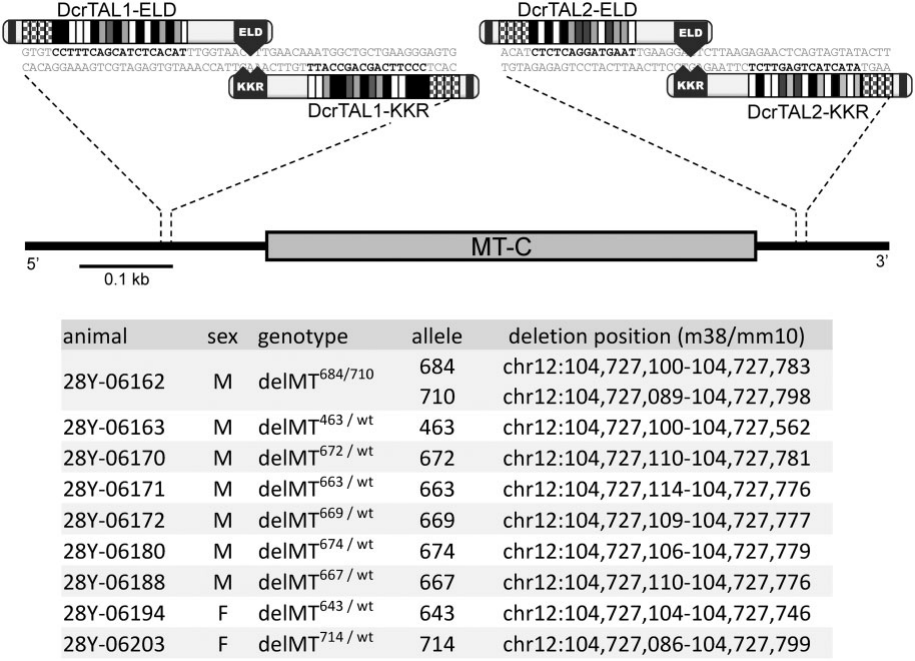
An example of genomic deletion achieved with two TALEN pairs [47]. Shown are relative positions of DSBs introduced by TALENs. Individual TALEN recognition sites are shown in bold black letters. Nine founder mice carrying 10 deletion alleles were found among 51 animals originating from TALEN-injected zygotes. Deletion positions are listed in the extreme right column.

TALEN technology was successfully used for targeted genome editing in yeasts [50], *Drosophila melanogaster* and other insect species [51–53], *Danio rerio* [34, 54], *Caenorhabditis elegans* [55], *Xenopus laevis* [56, 57], mouse [58, 59], rat [60] and livestock, including pig and cow [61]. Plants are also accessible for TALEN-mediated gene editing, including not only model organisms such as *Arabidopsis* [62, 63] but also crop plants such as rice [64] and tobacco [65]. Current efficiency varies usually from 10 to >50% with an average around 22% cells mutated [66, 67]. We achieved TALEN cleavage efficiency of 18% when inducing a ∼0.7 kb deletion with two TALEN pairs in the mouse genome [47] (Figure 4).

Early studies typically used NHEJ-mediated mutagenesis. DSB-driven HR with dsDNA donor templates was subsequently used as well, e.g. in human cells [28, 68] and zebrafish zygotes [69]. Single strand oligonucleotides with ∼50 nt long arms of homology were used as a donor template for precise modifications in zebrafish and mouse models [59,70]. Furthermore, introduction of two DSBs simultaneously allows for additional genome alterations [47, 52, 61]. A widely applied and generally successful approach is microinjection of *in vitro* synthesized mRNAs encoding a custom TALEN pair into the zygote [47, 59–61, 69, 70]. This allows for fast and effective preparation of knock-out models [71]. Heterozygous mutant mice can be prepared within 18 weeks [47, 59]. Biallelic mutations may also occur [47, 56, 61, 70], which strongly reduce time necessary for preparation of homozygous animals. We have produced and analyzed a knock-out mouse model within a year with frequency of genomic deletion of ∼20%; 1/51 founder animals carried the desired deletion on both chromosomes [47].

TALENs are highly specific and can distinguish sites, which differ only in two mismatched bases [13, 54]. Mussolino *et al.* [13] compared cytotoxicity and specificity of a CCR5-specific TALEN pair with a well-established ZFN pair. Off-target site in highly homologous CCR2 gene differed from CCR5 only in one base and 5′-T^0^. The TALEN pair induced only 1% mutation in the *CCR2* off-target, whereas the ZFN pair induced 11%. Moreover, 2-fold higher cell survival was reported for the TALEN pair. Numerous other results suggest that TALENs are more mutagenic and less cytotoxic than ZFN [34, 39, 66, 72, 73].

Enhanced TALEN-mediated gene disruption in rat zygotes was achieved by co-injection of engineered TALENs with Exonuclease 1 [74] or Trex exonuclease [75], which degrade one DNA strand in DSB site and therefore promote alternative mutagenic correction pathway [74]. Mutagenicity can be further improved by adoption of the more effective FokI nuclease such as *Sharkey* [76] or by transient hypothermia [28].

Superior TALEN specificity can be achieved by adopting a heterodimeric FokI architecture, by mutating cationic residues in TALE C-terminal domain [31], or via fusion with other cleavage domains with intrinsic sequence specificity such as meganucleases (MegaTALs) or TevI nuclease. Recently reported MegaTALs are compact, active and hyper-specific endonucleases valuable for future widespread, safe and reliable therapeutic use [75, 77]. TevI may work either as a monomeric nuclease (fused to N-terminus of TALE array over a TevI native linker) or as a nicking enzyme (fused to C-terminus of TALE array over shorter artificial linker), cleaving only one DNA strand [78]. The TevI cleavage domain (only ∼200 aa) has degenerated site specificity (CN↑NN↓GN), which limits possible target site selection, but substantially reduces the TALEN size [78]. Targeted nickases could be used to promote gene correction via HR in selected loci, with reduced cytotoxicity, because no DSBs are created [79, 80].

### Gene regulation with TALEs-DBDs

TALE DBDs were used not only for gene editing but also for targeted endogenous gene regulation in a form of artificial TALE transcription factors (Figure 3). The first study demonstrated activation of plant genes in *Arabidopsis* using a native AvrBs3 scaffold with designed CRD matching their promoters [33]. Zhang *et al.* [14] developed an artificial TALE activator (Figure 3B) using a truncated scaffold fused to the VP64 activation domain (tetrameric version of VP16 activation domain from *Herpes simplex* virus) and successfully induced expression of *SOX2* and *KLF4* in human cells but failed to activate *OCT4* and *c-MYC* genes [14]. Similarly, two other groups used different TALE architectures for activation of human genes with the VP16 domain [28, 81].

Activation of *Oct4* gene was achieved with a TALE-VP16 activator in murine embryonic stem cells and derived neural stem cells [82]. TALE-mediated gene activation seemed to depend on the binding-site position in a target promoter and consequent interactions with basal transcription factors. Authors also demonstrated that methylation of target promoters impairs TALE activity and that specific activation of silenced genes is possible once cells are treated with low concentration of histone deacetylases and/or DNA methyltransferases inhibitors [82]. Negative effects of DNA methylation on TALE binding can be solved by using NG and N* RVDs, which allow for accommodating 5′-methylcytosine [26, 83]. A set of human genes including non-coding microRNA cluster miR-302/367 was activated in another study, which also showed that using multiple TAL Effector based transcriptiont factors (TALE-TFs) targeting a single gene has a synergistic effect on target expression [84].

TALE fusions with effector domains offer a broad range of applications, ranging from simple locus-specific transcriptional activation and repression [82, 85], through direct induction of epigenetic changes on DNA [86] or on histones [87], to using them for visualization and pull-down of specific genomic loci [88–91].

### Design and assembly of TALE repeat domain

Several rules for rational design of TALE-CRD (and inherently for the selection of target site in DNA) could be inferred from known properties of particular repeat types and from the TALE-DNA-binding mode:

- Select target sites with 5′-T^0^ base preceding the CRD-specified sequence. If that is not possible, one can use reengineered scaffold with unrestricted specificity for 5′-N^0^ [38].
- Confirm that your selected target site is truly unique (e.g. not representing a unique polymorphism within a highly repetitive element).
- Although optimal repeat lengths likely vary for individual cognate sequences [31] as a rule of thumb [9, 48, 59], we recommend at least 14 repeats for each TALEN in a pair and 18–20 repeats for TALE transcription factors.
- Include at least four evenly positioned strong RVDs (e.g. HD > C, NG > T or NN > G/A), especially at termini of CRD to stabilize TALE-DNA interaction [27, 32]
- Avoid stretches of more than three identical RVDs, especially of NG, which was shown to adopt a deformed fold even with three repeats in a row [29].
- Use NH for targeting G instead of NN, if discrimination between A and G is necessary [29].
- Use NI for specific recognition of A along with sufficiently strong RVDs [29].
- Use validated TALE scaffold, which includes whole NTR (∼150 aa) and suitable C-terminal linker to the effector domain. One of the most common scaffolds established in multiple organisms is Miller’s [28]. Also Mussolino’s [13] and Zhang’s [14] architectures are reliable and were used repeatedly.
- Finally, we highly recommend to search for online tools for TALEN design and off-targeting analysis, which become increasingly available. Several of them are listed in Table 1.

**Table 1:** Selected TALEN design web tools

Tool	URL	References
E-TALEN	http://www.e-talen.org/E-TALEN/	[92]
tDnA	http://baolab.bme.gatech.edu/Research/BioinformaticTools/assembleTALSequences.html	[93, 94]
TALE-NT	https://tale-nt.cac.cornell.edu/node/add/talen	[95]
TALeffectors	http://taleffectors.genome-engineering.org/tools/	[96]
Mojo Hand	http://www.talendesign.org/	[97]

Because the assembly of designed TALE DBDs from nearly identical repeats was challenging for classical cloning techniques, several platforms have emerged for efficient and rapid (less than a week) construction of expression plasmids containing a TALE scaffold with a designed DBD (reviewed in [48]). A widely used platform is the ‘Golden Gate Cloning’, which allows for highly efficient assembly of designed TALEs in a single reaction [14, 62, 81, 98–100]. Recent advances in TALEN assembly methods include ligation-independent cloning [101] and solid-phase cloning such as Fast Ligation-based Automatable Solid-phase High-throughput platform for large scale assembly of TALENs (FLASH) [67] and Iterative Capped Assembly (ICA) [102], the latter allowing for a rapid automatized robotic assembly with a high-throughput capability. Needless to say, custom TALE nucleases are also available from numerous commercial sources.

## SUMMARY AND OUTLOOK

Simple design, fast and low-cost assembly, high specificity combined with low cytotoxicity and a practically unlimited target site selection make TALE DBDs an excellent choice for DNA targeting. The TALEN technology has superior mutagenic potential associated with lower cytotoxicity and higher target specificity compared with ZFNs. Simple design and publicly available assembly toolkits allow for adoption of this technology by laboratories worldwide. Modular nature of TALE-DNA recognition, no significant inter-repeat context effects in contrast to zinc fingers and a possibility to target practically any sequence in are other important features. Although TALENs currently face competition from recently developed RNA-guided clustered regularly interspaced short palindromic repeats (CRISPR) approach [103], their outstanding potential for research and therapy remains undisputed.

**Key points**

- TALEs interact with cognate sequences via tandem repeats, which bind individual nucleotides.
- A selected locus can be targeted with a designed TALE fused with an effector domain
- TALENs allow for genetic alterations in virtually any model system.
